# Mortality in Ventilator‐Associated Tracheobronchitis and Pneumonia in Oncology Patients: The Impact of Microbiological Aspects

**DOI:** 10.1155/cjid/5887462

**Published:** 2026-01-11

**Authors:** Vitor Falcao de Oliveira, Débora de Oliveira Lopes, Valdirene Santos Folli Cabral, Luiz Dalfior Junior, Simone Siqueira Matos, Luciana Alexandra Antônia de Almeida, Larissa Vasconcelos Barreto, João Manoel da Silva Junior, Ana Paula Cury, Odeli Nicole Encinas Sejas, Adriana Satie Gonçalves Kono Magri, Edson Abdala

**Affiliations:** ^1^ Department of Infectious Diseases and Tropical Medicine, University of Sao Paulo, Sao Paulo, São Paulo, Brazil, usp.br; ^2^ Cancer Institute of the State of São Paulo, University of Sao Paulo, Sao Paulo, São Paulo, Brazil, usp.br; ^3^ Centres for Antimicrobial Optimisation Network (CAMO-Net), Sao Paulo, São Paulo, Brazil; ^4^ Department of Central Laboratory, Microbiology Laboratory, University of Sao Paulo, Sao Paulo, São Paulo, Brazil, usp.br

**Keywords:** microbiology, multidrug resistance, oncology, ventilator-associated pneumonia, ventilator-associated tracheobronchitis

## Abstract

**Background:**

Most evidence on ventilator‐associated pneumonia (VAP)–related and ventilator‐associated tracheobronchitis (VAT)–related mortality comes from general ICU settings, with limited data on critically ill cancer patients. This study aimed to characterize the microbiological profile and resistance patterns in an oncology hospital and evaluate their impact on 14‐day mortality.

**Methods:**

We conducted a retrospective analysis of VAP and VAT cases in an oncology ICU in Brazil (Jan–Dec 2024), assessing bacterial frequency, multidrug‐resistant organisms (MDRO), and mortality. Multivariate analysis was used to identify the variables significantly associated with mortality.

**Results:**

Among 85 ICU patients, tracheobronchitis was more frequent (59%) than pneumonia (41%). Most were male (61%) with a median age of 62 years and had solid tumors (85%), mainly in the lungs and neck. Of 109 samples, *P. aeruginosa* (27%), *K. pneumoniae* (20%), and *S. maltophilia* (17%) were the most common pathogens. MDRO was particularly high in *A. baumannii* (82%). Overall, 14‐day mortality was 55%. MDR was not associated with mortality (*p* = 0.3), but VAP (OR 4.20, *p* = 0.004) and infections with positive blood culture (OR 5.38, *p* = 0.023) were independently associated with mortality.

**Conclusion:**

This study provides valuable insights into the microbiological profile of patients with VAP and VAT in an oncological ICU and its impact on mortality. Mortality was not associated with MDR, possibly reflecting the high baseline risk from underlying conditions. However, patients with positive blood cultures had significantly higher mortality, suggesting a more invasive disease.

## 1. Introduction

Ventilator‐associated pneumonia (VAP) and ventilator‐associated tracheobronchitis (VAT) often occur as a complication in critically ill patients [1–4]. In the context of healthcare‐associated infections, VAP is the most frequent and severe infection affecting patients hospitalized in intensive care units (ICUs) [5–7]. The incidence of VAP varies widely in the international literature, ranging from 8% to 30%. This variability reflects differences in patient profiles, duration of mechanical ventilation, diagnostic criteria, and surveillance contexts [2, 8–11].

The overall reported mortality from VAP ranges between 20% and 60%, in addition to its impact on prolonged hospitalization and increased cost per episode [12–14]. However, establishing a direct causal link is challenging due to the severity of the underlying conditions, particularly malignancies [8, 15].

Current scientific literature identifies several risk factors associated with increased mortality from VAP in oncology patients. These factors can be grouped into three main categories: clinical, microbiological, and treatment‐related [6, 16, 17]. From a microbiological perspective, infections caused by multidrug‐resistant (MDR) pathogens, particularly *Acinetobacter baumannii*, and the presence of bilateral pulmonary infiltrates are associated with worse outcomes.

Despite these findings, most of the available evidence on risk factors for VAP‐related mortality originates from studies conducted in general ICU populations and does not fully reflect the specificities of oncological patients. Individuals with cancer often present distinct clinical features, such as persistent immunosuppression and greater prior exposure to broad‐spectrum antimicrobials, which may significantly alter both microbiological profiles and clinical outcomes [16, 18].

Moreover, the high incidence of MDR pathogens in this group poses additional challenges for therapeutic management. A significant gap remains in the literature regarding the impact of these pathogens and their resistance patterns on short‐term mortality in critically ill cancer patients, highlighting the need for targeted studies in this population [16, 18]. Our objectives in this study were to characterize the microbiological profile of patients with VAP and VAT in oncological ICU and to assess the impact of the isolated microorganisms and their resistance pattern on 14‐day mortality.

## 2. Methods

### 2.1. Setting and Study Design

We performed a retrospective analysis of prospectively collected data from adult patients consecutively diagnosed with VAP and VAT in an oncological ICU in a Brazilian public hospital between January and December 2024.

Instituto do Câncer do Estado de São Paulo (ICESP) is a tertiary hospital with 490 beds and an ICU, with 85 beds, dedicated to adult oncological medical and surgical patients.

Ethical approval for this study was granted by the local committee. Confidential and sensitive data were removed, and all identification codes were anonymized. The ethics review board waived the requirement for informed consent.

### 2.2. Diagnosis and Definitions

The diagnosis of VAP and VAT was based on the CDC/NHSN diagnostic criteria, summarized in the supporting information (Table S1) [19, 20]. The bacteriologic diagnosis was obtained by quantitative culture using a positive threshold of 10^5^ and 10^4^ colony forming units (CFU/mL) for endotracheal aspirates and bronchoalveolar lavage (BAL) fluids, respectively. Cultures were considered as polymicrobial if > 1 microorganisms grew.

MDR organisms (MDRO) were defined as bacteria which exhibited resistance to one or more categories of antimicrobial drugs. These encompass methicillin‐resistant *Staphylococcus aureus* (MRSA), carbapenem‐resistant *Enterobacterales* (CRE), Gram‐negative bacteria producing extended‐spectrum ß‐lactamase, and organisms such as *Stenotrophomonas maltophilia* that are intrinsically resistant to the broadest‐spectrum antimicrobial agents [21].

Bacterial isolates were identified using matrix‐assisted laser desorption/ionization time of flight mass spectrometry (MALDI‐TOF MS) (bioMerieux). Antimicrobial susceptibility testing was performed using an automated system (Vitek 2 XL, bioMerieux) for the majority of isolates and complemented by disk diffusion for nonfermenting Gram‐negative bacilli, in accordance with the criteria and breakpoints established by the Clinical and Laboratory Standards Institute (CLSI). In cases of MDRO or discrepant results, additional confirmatory testing was performed as needed.

### 2.3. Collected Data

The following patient data were collected at diagnosis: age, sex, malignancies, duration of mechanical ventilation, ICU stay until the diagnosis of infection, culture of blood and respiratory samples (BAL or endotracheal aspirates), antimicrobial susceptibility test, severity score (SAPS III), appropriateness of initial antibiotic therapy, and mortality within 14 days.

Initial antibiotic therapy was considered appropriate when the bacteria identified in vitro were susceptible to at least one of the antibiotics administered within 48 h of the infection diagnosis. Empirical antimicrobials were selected based on the physician’s clinical judgment and internal ICU protocols.

### 2.4. Statistical Analysis

Analyses were performed using RStudio software Version 1.4. The frequency of bacteria, MDROs, types of malignancies, and deaths was assessed using total counts and percentages for categorical variables, while the median and interquartile range (IQR) were used for continuous variables, such as age and SAPS III.

We conducted comparisons between the survival and nonsurvival groups, as well as compared the clinical and microbiological characteristics of patients with VAT and VAP. For this analysis, categorical variables were assessed using Fisher’s exact test and Pearson’s chi‐squared test as appropriate, while the Mann–Whitney test was applied to continuous variables. Multivariable logistic regression analysis was performed to identify factors independently associated with mortality. The model included the type of malignancy, MDR status, and variables that were statistically significant in the group comparisons. Statistical significance was defined by a *p* value < 0.05.

## 3. Results

A total of 85 patients presented VAT and VAP during ICU stay. Tracheobronchitis was diagnosed in 50 cases (59%), while pneumonia was identified in 35 cases (41%). The majority was male (*n* = 52, 61%), and the median age was 62 years (IQR 53–70). VAT and VAP patient clinical characteristics are summarized in Table 1. Most patients had solid tumors (*n* = 72, 85%), primarily located in the neck (*n* = 11, 15%) and lungs/bronchi (*n* = 13, 18%). Among the hematological malignancies, almost half of the cases were lymphomas (*n* = 6).

**Table 1:** Demographic and clinical characteristics of 85 patients with ventilator‐associated tracheobronchitis and pneumonia in an oncological intensive care unit.

Characteristic	Number (%)
Age (years), median (IQR)	62 (53–70)
Male sex	52 (61)
Pneumonia	35 (41)
Tracheobronchitis	50 (59)
Solid tumor	72 (85)
Neck cancer	11 (15)
Mediastinal cancer	3 (4)
Breast cancer	6 (8)
Kidney cancer	2 (3)
Bladder cancer	2 (3)
Bone cancer	2 (3)
Central nervous system cancer	5 (6)
Prostate cancer	7 (10)
Uterus and ovarian cancer	5 (6)
Testicular cancer	2 (3)
Gastrointestinal cancer	7 (10)
Lung/bronchial cancer	13 (18)
Liver cancer	2 (3)
Undefined	2 (3)
Other	3 (4)
Hematological malignancy	13 (15)
Acute leukemia	3 (23)
Multiple myeloma	3 (23)
Lymphoma	6 (46)
Ambiguous phenotype	1 (8)
Multidrug‐resistant infection	30 (35)
14‐day mortality	47 (55)

We isolated 109 bacterial strains from these 85 respiratory infections, 22 of which (26%) were polymicrobial. The most common microorganisms were *Pseudomonas aeruginosa* (27%), followed by *Klebsiella pneumoniae* (20%) and *S. maltophilia* (17%) (Table 2). The most frequent microorganism combinations isolated in polymicrobial infections were *K. pneumoniae* + *P. aeruginosa* (*n* = 4), *P. aeruginosa* + *S. maltophilia* (*n* = 3), and *P. aeruginosa* + *S. aureus* (*n* = 3).

**Table 2:** Frequency of 109 microorganisms identified in samples collected from 85 patients with ventilator‐associated tracheobronchitis and pneumonia, the proportion of multidrug resistance and death for each bacteria.

Microorganisms	Total (*n* = 109) *n* (%)	MDR (%)	Death (%)
*Acinetobacter baumannii*	11 (10)	9 (82)	4 (36)
*Acinetobacter radioresistens*	1 (1)	0 (0)	1 (100)
*Atopobium rimae*	1 (1)	0 (0)	0 (0)
*Enterobacter cloacae* complex	4 (4)	2 (50)	1 (25)
*Escherichia coli*	2 (2)	1 (50)	2 (100)
*Klebsiella aerogenes*	1 (1)	0 (0)	0 (0)
*Klebsiella pneumoniae* complex	22 (20)	14 (64)	13 (59)
*Proteus mirabilis*	1 (1)	0 (0)	0 (0)
*Pseudomonas aeruginosa*	29 (27)	10 (38)	17 (58)
*Serratia marcescens*	2 (2)	0 (0)	2 (100)
*Staphylococcus aureus*	15 (14)	4 (27)	9 (60)
*Stenotrophomonas* *maltophilia*	19 (17)	100 (19)	12 (63)
*Streptococcus pneumoniae*	1 (1)	0 (0)	1 (100)

Fifty‐nine (54%) of the bacterial isolates were classified as MDR. Among the MDROs, the bacteria with higher proportion of resistance, apart from *Stenotrophomonas*, were *A. baumannii* (82%), *K. pneumoniae* (64%), *Enterobacter cloacae* complex (50%), and *Escherichia coli* (50%). All cases of *S. maltophilia* were susceptible to trimethoprim–sulfamethoxazole and levofloxacin.

Ninety‐five microorganisms were isolated from respiratory samples, with a predominance of Gram‐negative bacteria, including *P. aeruginosa*, *S. maltophilia*, and *K. pneumoniae* as the most frequent (Figure 1(a)). The main type of sample collected was tracheal aspiration (*n* = 93, 98%), while only two patients underwent BAL sampling.

Figure 1Frequency of microorganisms isolated from samples of patients with ventilator‐associated tracheobronchitis and pneumonia. (a) Respiratory samples and (b) blood samples.

**Figure:**
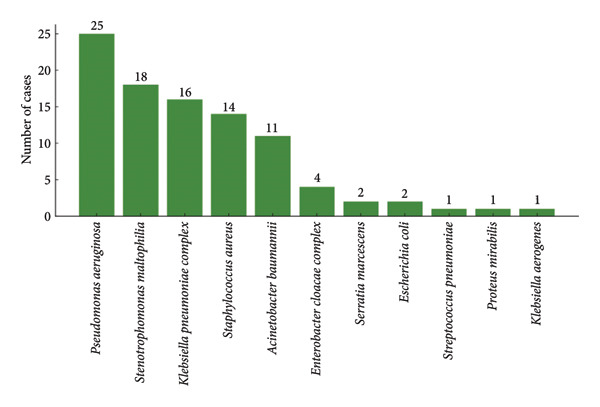
(a)

**Figure:**
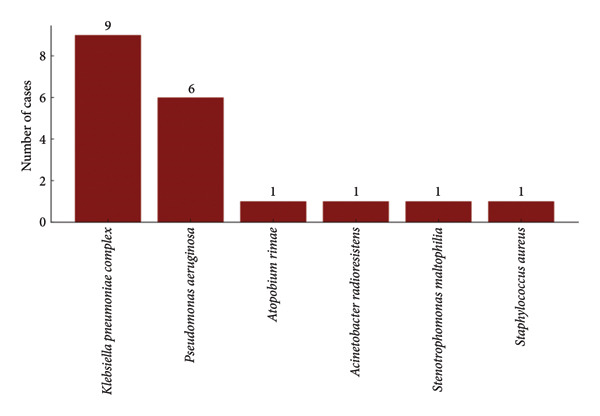
(b)

Nineteen microorganisms were isolated from blood samples, also predominantly Gram‐negative, with *K. pneumoniae* being the most common (*n* = 9, 50%) (Figure 1(b)). The same microorganisms were isolated in blood and respiratory in four patients.

**Figure 2:**
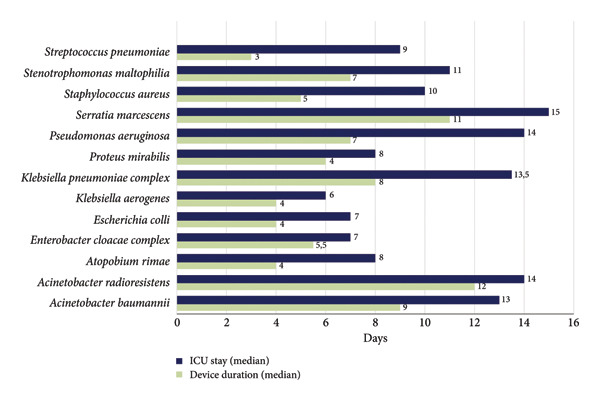
Device duration and intensive care unit length of stay by microorganism for patients with ventilator‐associated tracheobronchitis and pneumonia.

Patients infected with *S. maltophilia*, *Acinetobacter* spp., *Serratia marcescens*, *P. aeruginosa*, and *K. pneumoniae* presented longer hospital stays, with a median duration of more than 10 days. Device use was also prolonged in patients with infections caused by *S. marcescens*, *K. pneumoniae*, and *Acinetobacter* spp., with a median duration exceeding 7 days (Figure 2). Additionally, regarding the type of airway device, 27% (23/85) of patients had a tracheostomy, while 73% (62/85) were intubated with an orotracheal tube.

The 14‐day mortality was 55% (47/85). Evaluating the deaths according to isolated microorganisms, we detected the highest rates associated to *Acinetobacter radioresistens* (*n* = 1, 100%), *E. coli* (*n* = 2, 100%), *P. aeruginosa* (*n* = 17, 58%), *S. marcescens* (*n* = 2, 100%), *Streptococcus pneumoniae* (*n* = 1, 100%), *S. maltophilia* (*n* = 12, 63%), *S. aureus* (*n* = 9, 60%), and *K. pneumoniae* complex (*n* = 13, 59%) (Table 2).

**Figure 3:**
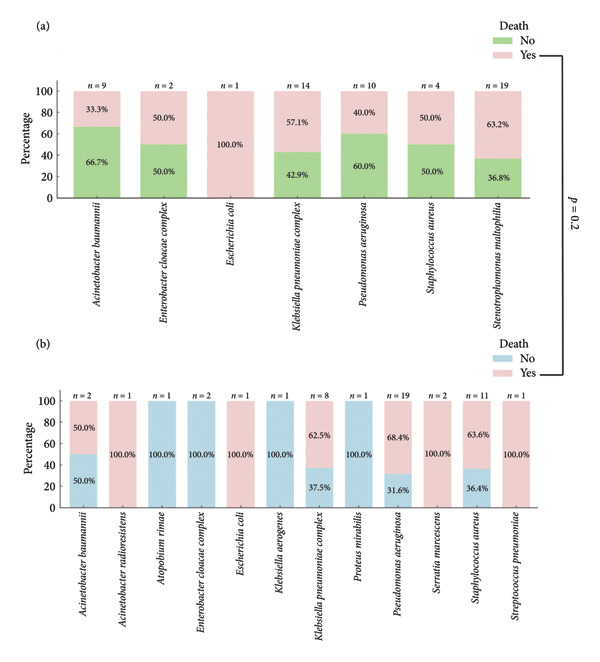
Frequency of mortality within 14 days of hospitalization related to ventilator‐associated tracheobronchitis and pneumonia according to isolated microorganism. (a) MDR infection and (b) non‐MDR infection.

Nonsurvivors and survivors showed similar distributions of age, sex, type of malignancy, SAPS III score, bacterial prevalence, and appropriateness of initial antibiotic therapy. Notably, there was no significant association between MDR and mortality (*p* = 0.2) (Figure 3 and Table 3). Mortality was 47% (14/30) among patients with MDR infections and 60% (33/55) among those with non‐MDR infections. However, patients with positive blood cultures had significantly higher mortality compared to those with only respiratory samples (28% versus 8%, *p* = 0.02).

**Table 3:** Comparison of survivor and nonsurvivor patients with ventilator‐associated tracheobronchitis and pneumonia in an oncological intensive care unit.

Characteristic	Survivors (*n* = 38)	Nonsurvivors (*n* = 47)	*p* value
Age (years), median (IQR)	61 (44–69)	62 (55–70)	0.4
Male sex	22 (59%)	30 (65%)	0.6
Respiratory tract infection			**0.003**
Pneumonia	9 (24%)	26 (55%)	
Tracheobronchitis	29 (76%)	21 (45%)	
Type of malignancy			> 0.9
Solid tumor	32 (84%)	40 (85%)	
Hematological malignancy	6 (16%)	7 (15%)	
Positive blood culture	3 (8%)	13 (28%)	**0.02**
SAPS III, median (IQR)	63 (48–73)	63 (54–71)	0.8
Multidrug resistant	16 (42%)	14 (30%)	0.2
Bacteria			
*Acinetobacter baumannii*	4 (11%)	2 (4%)	0.4
*Klebsiella pneumoniae*	8 (21%)	8 (17%)	0.6
*Pseudomonas aeruginosa*	7 (18%)	7 (15%)	0.7
*Staphylococcus aureus*	2 (5%)	4 (9%)	0.7
*Stenotrophomonas maltophilia*	6 (16%)	9 (19%)	0.7
Polymicrobial	8 (21%)	14 (30%)	0.4
Appropriateness of initial antibiotic therapy	21 (55%)	23 (49%)	0.6

*Note:* Bold values indicate statistical significance (*p* < 0.05).

In the comparison between patients with VAP and VAT, mortality was significantly higher in the VAP group (74% vs. 42%, *p* = 0.003). In contrast, the prevalence of MDR pathogens, the distribution of bacterial species, the median duration of device use, and ICU length of stay were similar between the groups, with no statistically significant differences (Table S2).

In the multivariate analysis, positive blood cultures and VAP were analyzed separately because both variables showed high collinearity based on the diagnostic criteria (all patients with positive blood cultures had VAP). Positive blood cultures (OR 5.38, 95% CI: 1.43–28.1, *p* = 0.023) and VAP (OR 4.20, 95% CI: 1.63–11.7, *p* = 0.004) were independently associated with mortality, whereas MDR infection was not associated with mortality in either model (*p* = 0.3) (Table 4).

**Table 4:** Variables associated with mortality by multivariate analyses in patients with ventilator‐associated tracheobronchitis and pneumonia in an oncological intensive care unit.

Characteristics	Model 1			Model 2		
	OR	95% CI	*p* value	OR	95% CI	*p* value
Pneumonia	4.20	1.63–11.7	**0.004**	—	—	—
Malignancy	1.54	0.41–5.85	0.5	1.82	0.47–7.91	0.4
Positive blood culture	—	—	—	5.38	1.43–28.1	**0.023**
Multidrug resistant	0.61	0.23–1.58	0.3	0.6	0.23–1.51	0.3

*Note:* Bold values indicate statistical significance (*p* < 0.05).

## 4. Discussion

Mortality among oncological patients with VAP and VAT was high, exceeding 50%, primarily associated with Gram‐negative bacteria isolated from respiratory and blood samples. Patients with positive blood cultures had higher mortality than those with only respiratory samples, although this was not significantly associated with the MDRO status. *A. baumannii* showed the highest resistance, but mortality was greater among patients infected with *S. maltophilia* or *S. aureus*.

As observed in our study, respiratory tract infections are most frequent in patients with leukemia/lymphoma and in those with lung or neck solid tumors [22]. VAP and VAT are predominantly caused by bacteria, while fungi and viruses are infrequent etiological agents [23]. In oncology patients, the microbial etiology of these infections reflects an immunocompromised status, prolonged use of invasive devices, and frequent prior exposure to broad‐spectrum antimicrobials [18]. The most commonly isolated pathogens in ICU‐acquired pneumonia include especially Gram‐negative bacteria such as *K. pneumoniae* and *P. aeruginosa*, as well as Gram‐positive organisms such as *S. aureus* [8, 23–26]. According to Canadian guidelines, *S. aureus* and *P. aeruginosa* are the most frequently isolated organisms in adults with nosocomial pneumonia from ICU settings [27]. Interestingly, *S. maltophilia* was the third most common pathogen in our study, which is unusual compared to other series in nononcological populations. It has been described that the primary predisposing factor for *S. maltophilia* infection is an immunocompromised status [27, 28].

Positive blood cultures range from 0% to 40% as reported in the AMMI Canada guidelines for hospital‐acquired pneumonia and VAP [29]. A previous study demonstrated that blood cultures had a higher proportion of Gram‐positive bacteria for healthcare‐associated pneumonia, such as *S. aureus* and *S. pneumoniae*, compared to respiratory samples, which showed a lower proportion of Gram‐negatives (55% vs. 36%) [30]. However, our study revealed that even in blood cultures, Gram‐negative bacteria were predominant. In fact, the proportion of patients with Gram‐positive bacteria was actually higher in respiratory samples than that in blood cultures. In the same study, mortality was higher among patients with positive blood cultures [30], a finding also observed in our study.

VAP and VAT can be polymicrobial in up to 30% of cases, which is consistent with our findings (26%). The increasing prevalence of MDRO further highlights the importance of early risk stratification and the appropriate selection of empirical antimicrobial therapy [5, 31], due to its association with higher mortality for VAP. The ICU 30‐day mortality rate ranged from 31.9% to 66.7% for *A. baumannii*, particularly for MDR [32]. In our study, *A. baumannii* showed the highest proportion of multidrug resistance. Consistently, more than 70% of the patients who died had infections caused by MDR. However, we found no association between MDR and mortality across all microorganisms. This likely reflects the impact of underlying conditions, particularly malignancies, which on their own can explain the higher mortality.

This study has some limitations. Being a single‐center and retrospective study, its findings may have limited generalizability, particularly to ICUs with different pathogen profiles or institutional practices. Mortality may have been influenced not only by the bacterial profile but also by factors not assessed in our analysis, such as the stage of oncological treatment. Additionally, some bacteria had small sample sizes in this study, which may have overestimated the mortality. Further studies are needed to better understand the microbiological factors associated with mortality in this oncology population.

## 5. Conclusion

This study provides valuable insights into the microbiological profile of patients with VAP and VAT in an oncological ICU and its impact on mortality. Gram‐negative bacteria were predominant, including in blood cultures, with *S. maltophilia* emerging unexpectedly as one of the most common pathogens. Mortality was not associated with MDR, as *S. maltophilia* showed high mortality, more than *A. baumannii*, despite being fully susceptible to trimethoprim–sulfamethoxazole and levofloxacin. However, patients with positive blood cultures and those with VAP had significantly higher mortality, suggesting a more invasive disease.

## Disclosure

All authors reviewed and approved the final manuscript.

## Conflicts of Interest

The authors declare no conflicts of interest.

## Author Contributions

Vitor Falcao de Oliveira, Odeli Nicole Encinas Sejas, and Edson Abdala wrote the main manuscript text. Débora de Oliveira Lopes, Valdirene Santos Folli Cabral, Larissa Vasconcelos Barreto, Odeli Nicole Encinas Sejas, Simone Siqueira Matos, and Luciana Alexandra Antônia de Almeida collected the data. João Manoel da Silva Junior, Ana Paula Cury, and Adriana Satie Gonçalves Kono Magri helped with the data curation and formal analysis. Vitor Falcao de Oliveira prepared all figures and tables.

## Funding

This research received no external funding.

## Supporting Information

Supporting Information associated with this article is available online and provides additional tables supporting the definitions and findings of this study.

## Supporting information


**Supporting Information** Additional supporting information can be found online in the Supporting Information section.

## Data Availability

The data that support the findings of this study are available from the corresponding author upon reasonable request.
