# Supplementary material for: Mortality in Ventilator‐Associated Tracheobronchitis and Pneumonia in Oncology Patients: The Impact of Microbiological Aspects
Source: Can J Infect Dis Med Microbiol. 2026 Jan 11;2026:5887462. doi: 10.1155/cjid/5887462 (PMC12791571; doi:10.1155/cjid/5887462)
Supplement: Supplementary file 1 — Supporting Information Additional supporting information can be found online in the Supporting Information section. [file CJID-2026-5887462-s001.docx]

**Supplementary Material**

**Oliveira VF *et al*.** Mortality in Ventilator-Associated Tracheobronchitis and Pneumonia in Oncology Patients: The Impact of Microbiological Aspects

| Table S1 | Pages 2-3 |
| --- | --- |
| Table S2 | Pages 4-5 |

**Table S1.** Diagnostic criteria for ventilator-associated tracheobronchitis and pneumonia based on CDC/NHSN diagnostic criteria

| **Criteria** | **Pneumonia** | **Tracheobronchitis** |
| --- | --- | --- |
| And at least one of the following items: | | |
| **Mechanical ventilation** | Mechanical ventilation > 2 consecutive calendar days on the date of infection, with day of ventilator placement being Day 1, and the ventilator was in place on the date of event or the day before | |
| **Radiological findings** | - New or progressive and persistente infiltrate * - Consolidation - Cavitation | There is no radiological evidence of pneumonia |
| **Clinical and laboratory findings** | - Fever (temperature >38°C) with no other cause - Leukopenia (≤4,000 WBC/mm³) or leukocytosis (≥12,000 WBC/mm³) - Altered mental status with no other cause in ≥ 70 years old | At least 2 of the following signs or symptoms with no other recognized cause:   - Fever (>38°C) - Cough - New or increased sputum production - Rhonchi - Wheezing |
| **Pneumonia diagnostic signs/symptoms** | - New onset of purulent sputum, or change in character of sputum, or ↑ respiratory secretions, ↑ suctioning requirements - New onset or worsening cough, or dyspnea, or tachypnea - Rales or bronchial breath sounds (wheezing, rhonchi, or crackles on auscultation) - Worsening gas exchange, such as oxygen desaturation, ↑ oxygen requirements, or ventilation demand | Not applicable |
| **Microbiological findings** | - Positive blood culture not realted to another infection - Positive pleural fluid culture - Positive quantitative culture from minimally contaminated low respiratory tract specimen (e.g., bronchoalveolar lavage or protected specimen brushing) | - Positive culture obtained by deep tracheal aspirate or bronchoscopy |

* Patients with underlying diseases had 2 or more serial X-rays, whereas those without underlying diseases had at least 1 serial X-ray.

**Table S2.** Comparison of clinical and microbiological characteristics among ventilator-associated tracheobronchitis and pneumonia in an oncological intensive care unit

| **Characteristics** | **Pneumonia**  (n=35) | **Tracheobronchitis**  (n = 50) | **p-value** |
| --- | --- | --- | --- |
| **Age (years), median (IQR)** | 61 (48 - 69) | 64 (55 - 73) | 0.3 |
| **Male sex** | 25 (74%) | 27 (55%) | 0.088 |
| **SAPS III, median (IQR)** | 61 (48 - 69) | 64 (55 - 73) | 0.3 |
| **Type of malignancy** |  |  | 0.11 |
| Solid tumors | 27 (77%) | 45 (90%) |  |
| Hematological malignancy | 8 (23%) | 5 (10%) |  |
| **Appropriateness of initial antibiotic therapy** | 21 (60%) | 23 (46%) | 0.2 |
| **Positive blood culture** | 16 (46%) | 0 (0%) | **< 0.001** |
| **Mortality** | 26 (74%) | 21 (42%) | **0.003** |
| **Multidrug-resistant infection** | 11 (31%) | 19 (38%) | 0.5 |
| **Bacteria** | | | |
| *Klebsiella pneumoniae complex* | 10 (29%) | 8 (16%) | 0.2 |
| *Pseudomonas aeruginosa* | 11 (31%) | 11 (22%) | 0.3 |
| *Staphylococcus aureus* | 1 (3%) | 8 (16%) | 0.075 |
| *Stenotrophomonas maltophilia* | 6 (17%) | 10 (20%) | 0.7 |
| *Enterobacter cloacae complex* | 0 (0%) | 3 (6%) | 0.3 |
| Median of device duration, days (IQR) | 7 (5 - 10) | 7 (5 - 13) | 0.5 |
| Median of intensive care unit length of stay, days (IQR) | 13 (10 - 18) | 10 (7 - 18) | 0.2 |
